# Ethylene Response Factor MaERF012 Modulates Fruit Ripening by Regulating Chlorophyll Degradation and Softening in Banana

**DOI:** 10.3390/foods11233882

**Published:** 2022-12-01

**Authors:** Hangcong Chen, Xiuhua Lai, Lihua Wang, Xueping Li, Weixin Chen, Xiaoyang Zhu, Zunyang Song

**Affiliations:** 1 Guangdong Provincial Key Laboratory of Postharvest Science of Fruits and Vegetables, Engineering Research Center for Postharvest Technology of Horticultural Crops in South China, Ministry of Education, College of Horticulture, South China Agricultural University, Guangzhou 510642, China; 2 Key Laboratory of Food Processing Technology and Quality Control in Shandong Province, College of Food Science and Engineering, Shandong Agricultural University, Tai’an 271018, China

**Keywords:** chlorophyll degradation, ‘Fenjiao’ banana, MaERF012, starch and cell degradation, transcription factor, yellowing

## Abstract

Ethylene response factors (ERFs) are one of largest plant-specific transcription factor families involved in fruit ripening. However, the regulatory mechanism by which ERFs modulate fruit yellowing and softening remains unknown in banana. We previously found that the transcription of *MaERF012* was closely related to ‘Fenjiao’ banana fruit ripening. Herein, we found that *MaERF012* was differentially expressed in the fruit pulp and peel and was closely related to fruit ripening. MaERF012 activated the promoter activity of one chlorophyll degradation gene (*MaSGR1*), two starch degradation genes (*MaGWD1* and *MaAMY3*), and three cell wall degradation genes (*MaPL8*, *MaEXP-A8*, and *MaXYL23-like*), which were tested by EMSA, Y1H, and DLR. Transient overexpression of *MaERF012* accelerates fruit ripening by promoting fruit yellowing and softening by up-regulating the transcription of chlorophyll, starch, and cell wall degradation genes. Over-expression of *MaERF012* alters the transcriptome profiles of the fruit peel and pulp, and the differentially expressed genes were mainly enriched in starch and sucrose metabolism, plant hormone signal transduction, biosynthesis of secondary metabolism, and fructose and mannose metabolism. Overall, the data showed that MaERF012 acts as a transcriptional activator by regulating fruit ripening by activating the transcription of chlorophyll, starch, and cell wall degradation genes.

## 1. Introduction

Banana (*Musa acuminata* L.) fruit ripening is a complicated biochemical and physiological process that causes physical changes including color change (from green to yellow), softening [1], and the appearance of aromatic substances [2]. Chlorophyll (Chl) degradation is a common natural occurrence during the ripening of green to yellow fruit peels [3], which is a multi-step enzymatic process [4]. The mechanism of Chl degradation has been well reported in plants [5]; however, few previous studies have reported the regulation of Chl degradation in fruit. In apple, MdPUB24 modulates ubiquitination of MdBEL7 to accelerate Chl degradation by activating the promoter activity of *MdCLH*, *MdPPH2*, and *MdRCCR2* [4]. In lychee, LcABF1/2/3 promotes fruit Chl degradation by increasing the expression of *LcSGR*, *LcPAO*, *LcCLH*, and *LcNYC* [5]. Previous studies on banana have identified many nonfluorescent Chl catabolites (NCCs), fluorescent catabolites (FCCs), and stay-green protein (SGR) genes related to Chl degradation [3,6,7]. Additionally, MaLUL2 inhibited fruit Chl degradation by repressing the transcription of *MaNYC1*, *MaSGR1*, *MaSGR2*, *MaPPH*, and *MaPAO* [8]. Despite these findings, the full regulatory mechanism for Chl degradation in banana is not clear.

Softening also plays a vital role in fruit quality and is related to cell wall and starch degradation [1,9]. In apple, 31 genes related to cell wall degradation have been identified [10]. A total of 38 and 24 starch degradation genes have been found in banana and kiwifruit, respectively [1,11]. Previous studies identified 61 cell wall and 38 starch degradation genes in ‘Fenjiao’ banana [3,12]. Additionally, cell wall and starch degradation were found to be regulated by transcription factor SlHY5 (elongated hypocotyl 5), which modulates starch degradation by interacting with the promoters of *SlDPE1*, *SlMEX1*, *SlBAM8*, *SlBAM3*, *SlBAM1*, and *SlPWD* in tomato [13]. In banana, MaMYB3 interacts with MabHLH6 and cooperatively mediates starch degradation by directly binding their promoters [14]. MaHDZII.4, MaHDZII.7, MaHDZI.19, and MaHDZI.26 up-regulated the expression of cell wall degradation genes [15]. In ‘Fenjiao’ banana, our previous research showed that MaABI5-like, MaC2H2-like, and MaNAC67-like proteins interact with MaEBF1 to modulate fruit ripening and involved in regulating a fruit softening disorder by activating the transcription level of cell wall and starch degradation genes [3,9,16]. However, the regulation of fruit softening in ‘Fenjiao’ bananas is still largely unknown.

Ethylene is an important endogenous hormone that triggers the onset of the ripening process of most fruits, especially climacteric fruit such as banana [9]. Ethylene response factors (ERFs), which are involved in regulating fruit ripening, belong to the AP2/ERF family. In tomato, the overexpression of *SlERF.B3* represses the onset of ripening, improves ethylene production, and promotes fruit softening [17,18]. In sweet orange, CitERF71 modulates the synthesis of volatile compounds by activating the expression of the terpene synthase gene *CitTPS16* [19]. In papaya, CpERF9 represses the degradation of the cell wall by inhibiting the expression of *CpPG5* and *CpPME1/2* [20]. PpeERF2 modulates fruit softening by repressing the transcription of *PpePG1* in peach [21]. In pear, PpERF24 modulates fruit ripening by modulating *PpACO54* expression [22]. In durian fruit, DzERF6 and DzERF9 may regulate fruit ripening by mediating ethylene production [23]. In banana, MaERF9 and MaERF11 interact with the promoters of *MaACS1* and *MaACO1*, respectively, to mediate ethylene production [24]. All of these results show the important role of ERFs in fruit ripening.

‘Fenjiao’ banana (*Musa* ABB Pisang Awak) is widely cultivated in southern China due to its excellent flavor and tolerance to environmental stressors [25]. ‘Fenjiao’ banana is different from the commonly consumed ‘Baxi’ banana (*Musa acuminata* L. AAA group cv. Cavendish), specifically in terms of ethylene synthesis and starch degradation characteristics; thus, ‘Fenjiao’ banana has higher sugar content and a unique fragrance [26]. The postharvest physiology of ‘Fenjiao’ banana is also dramatically different from ‘Baxi’ banana; ‘Fenjiao’ ripens and softens more rapidly than ‘Baxi’ banana [27]. Our previous study found that treatment with 1-MCP can inhibit fruit ripening, but a high concentration of 1-MCP caused a ripening disorder in which the fruit ripened with a softened green peel [3]. Moreover, the expression of MaERF012 was closely associated with a ripening disorder. Herein, we further studied the function of MaERF012 in the fruit ripening process, with a focus on the downstream target genes and the regulatory network. The results provide new insights regarding the ERF TF family’s role in regulating fruit yellowing and softening.

## 2. Materials and Methods

### 2.1. Plant Materials, Treatments, and Physiological Index Measurements

‘Fenjiao’ bananas (*Musa* ABB Pisang Awak cv. ‘Guangfen NO.1′) were harvested at the mature green stage with a plumpness of 85% to 90% from a farm in Qingyuan City, Guangdong Province, China and rapidly transported to a laboratory. The fruit were manually separated into individual fingers, and fruit with uniform maturity and without visual defects were selected. The bananas were then dipped in a 0.2% (*w*/*v*) hypochloride solution for 10 min and soaked in a 500 µL·L^−1^ mixture of iprodione (Huifeng, Yangzhou, China) and prochloraz (Kuaida, Yangzhou, China) for 1 min. 

**Figure 1:**
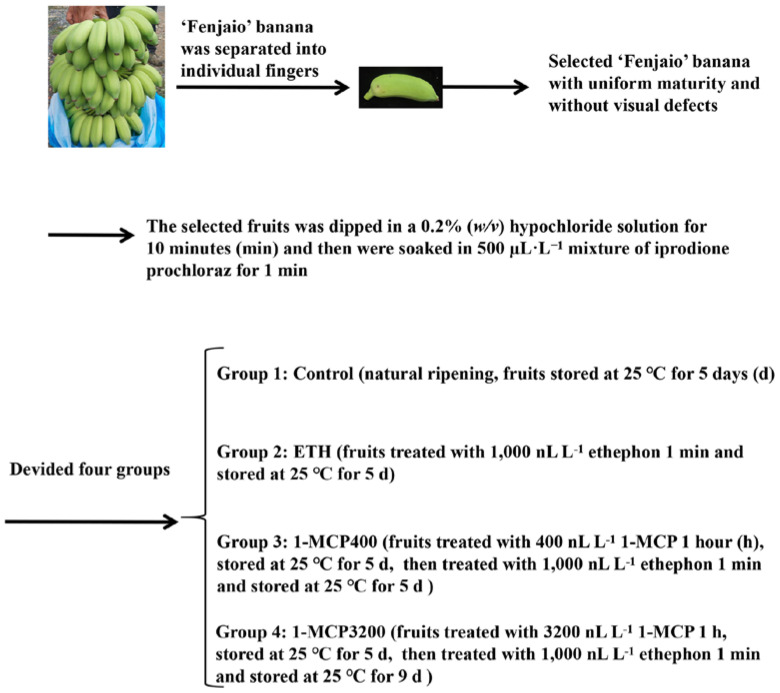
The treatment of ‘Fenjiao’ banana.

The selected pretreated fruit were split into four groups randomly. Control (natural ripening) and ethephon treatment (1000 nL·L^−1^ ethephon (ETH) solution for 1 min immersion) groups each contained 180 fingers of bananas, which were divided into 18 subgroups (each with 10 fingers) and stored separately in chambers at 25 °C for ripening. The suitable 1-MCP treatment group (400 nL·L^−1^ for 1 h (1-MCP400)) and the high-concentration 1-MCP treatment group (3200 nL·L^−1^ for 1 h (1-MCP3200)) each contained 270 fingers of bananas, which were similarly split into 27 subgroups (each with 10 fingers) and stored separately in chambers at 25 °C. After 5 days of storage, the fruit were treated with 1000 nL·L^−1^ ethephon (ETH) solution for 1 min immersion and then stored at 25 °C for ripening. This group’s fruit were collected at 0 d, 0.25 d (6 h), 1 d, 3 d, and 5 d after storage. Moreover, the 1-MCP400 group was also collected at 1, 3, and 5 d after ethephon treatment and the 1-MCP3200 group was collected at 1, 3, 5, 7, and 9 d after ethephon treatment (Figure 1). 

The color index was evaluated using the fruit ripening index, which was assessed on a scale from 1 to 7 as described by Zhu et al. [28]. For ethylene production, three fruit were weighed and individually placed in an airtight container equipped with a rubber topper for 2 h at 25 °C, and triplicate samples of 1 mL of headspace gas were taken for ethylene production according to the method of Zhu et al. [28]. Fruit and pulp firmness was measured according to the methods described by Zhu et al. [28]. Fruit firmness (N) was expressed as the mean of three measurements.

### 2.2. RNA Extraction and Transcript Analysis

RNA was extracted from ‘Fenjiao’ banana fruit peels and pulp using an RNA Extraction Kit (Aidlab Biotechnologies Co., Ltd., Beijing, China) and then reversed to cDNA using a Reverse Transcription Kit (Takara, Japan). RT-qPCR was conducted according to Song et al. [12]. The reaction system contained 10 μL of SYBR Premix Ex Taq, 1 μL of upstream and 1 μL of downstream primers, 100 ng of cDNA, and a volume of sterile deionized water leading to a final volume of 20 μL. The same set of reaction conditions was for all primers: 95 °C for 30 s, followed by 40 cycles at 95 °C for 5 s, 60 °C for 15 s, and finally 72 °C for 20 s. The expression levels of genes on day 0 were set as the calibrator. *MaACTIN* was set as the reference gene for the expression assay [29].

### 2.3. Subcellular Localization 

Subcellular location was analyzed according to Ding et al. [30]. The full coding sequence (CDS) of *MaERF012*, excluding the stop codon, was inserted into the pEAQ-GFP vector, which was stored in our laboratory, and a pEAQ-GFP vector with no insert was used as the control. The MaERF012-pEAQ-GFP and pEAQ-GFP vectors were separately transformed in the *Agrobacterium tumefaciens* strain GV3101, and the MaERF012-pEAQ-GFP and pEAQ-GFP vectors were then allowed to separately infiltrate into *N. benthamiana* leaves. After 36 h of infiltration, the subcellular location was detected using a universal fluorescence microscope (Zeiss Axioskop 2 Plus, Zeiss, Oberkochen, Germany). All transient expression assays were repeated at least three times.

### 2.4. Dual-Luciferase Transient Expression (DLR) Assays 

ERF TFs regulate download target genes by binding the GCC-box motif in their promoters [31]. Several Chl, cell wall, and starch degradation genes were identified in our previous work, and we found that the promoter sequence of four Chl degradation-related genes (*MaSGR1*, *MaNYC*, *MaPPH*, and *MaNOL*), four cell wall degradation-related genes (*MaPL8*, *MaEXP-A8*, *MaSUR14-like*, and *MaXYL23-like*), and three starch degradation-related genes (*MaBAM3*, *MaGWD1*, and *MaAMY3*) contained the GCC-box motif. These genes were individually transformed into the pGreenII 0800-LUC vector as a reporter, and the CDS of *MaERF012* was transformed into the pGreenII 62-SK vector as an effector. The pGreenII 0800-LUC and pGreenII 62-SK vectors were stored in our laboratory. The effector and reporter plasmids were co-infiltrated into *N. benthamiana* leaves. After 48 h of infiltration, DLR was detected according to the method of Song et al. [12]. Briefly, a DLR Assay kit (Promega, Madison, WI, USA) was used, and at least six independent replicates were conducted for each combination.

### 2.5. Yeast One-Hybrid (Y1H) Assay

Y1H assays were carried out using the Matchmaker Gold Yeast One-Hybrid System (Clontech, Fitchburg, WI, USA). The promoters of *MaSGR1*, *MaGWD1*, *MaAMY3*, *MaPL8*, *MaEXP-A8*, and *MaXYL23-like* were cloned into pAbAi, which was stored in our laboratory. It was then linearized and separately introduced into the Y1H Gold strain. The CDS of *MaERF012* was ligated to the pGADT7 vector and subsequently introduced into the yeast strain. It was then transferred into the aforementioned bait reporter yeast strain containing the promoters of *MaSGR1*, *MaGWD1*, *MaAMY3*, *MaPL8*, *MAEXP-A8*, and *MaXYL23-like*. Their interaction was tested according to the method of Song et al. [12].

### 2.6. Electrophoretic Mobility Shift Assay (EMSA)

The CDS of *MaERF012*, excluding the stop codon, was cloned into the pGEX-4T-1 vector (which was stored in our laboratory) to obtain the GST-MaERF012 recombinant plasmid and was then transformed into BM Rosetta (DE3) cells following the method of Song et al. (2022). The GST-MaERF012 protein was induced by 0.5 mM isopropyl-β-D-thiogalactopyranoside (IPTG) (Car. no. 9030; TaKaRa, Tokyo, Japan) at 28 °C for 6 h and purified using a GST Purification Kit (Cat. No. 635619, Clontech, Takara, Dalian, China) according to the manufacturer’s protocol.

The probes that contained the GCC-box (GCCGCC/GGCGGC) sequence derived from the promoters of the Chl degradation-related gene (*MaSGR1*), two starch degradation-related genes (*MaGWD1* and *MaAMY3*), and three cell wall degradation genes (*MaPL8*, *MaEXP-A8*, and *MaXYL23-like*) were labeled with biotin using the PierceTM Biotin 3′ End DNA Labeling Kit (Cat. No. 89818; Thermo Fisher Scientific, Waltham, MA, USA). An EMSA was conducted using the Light Shift Chemiluminescent EMSA Kit (Cat: No. 20148, Thermo Fisher Scientific, Waltham, MA, USA) as described by a previous study [12].

### 2.7. Transient Overexpression of MaERF012 in ‘Fenjiao’ Banana Fruit and the RNA-Seq Assay 

The CDS of *MaERF012* was cloned into the pMDC32 vector, which was stored in our laboratory. The ‘Fenjiao’ banana fruits were then infected as described previously [9]. The fruits were stored at 22 °C and relative humidity was set at 90%. The ethylene production, color index, firmness, and transcript level were tested and collected according to methods described above.

The empty vector and OE-MaERF012 banana fruit pulp and peel samples collected on day 3 were further investigated via RNA-Seq analysis using an Illumina HiSeq platform. Triple biological replicates were used for each sampling time point. Clustered profiles with a fold-change of ≥2 and a *p*-value ≤ 0.05 were considered to be differentially expressed genes (DEGs). A heat map was drawn using TBtools [32].

### 2.8. Data Analysis

All experiments were conducted in triplicate or six times in a completely randomized design. The results are presented as the mean ± SD using three or six independent biological replicates; statistical significance was analyzed with ANOVA followed by Duncan’s multiple range test using SPSS version 16.0 (SPSS, Inc., Chicago, IL, USA). Charts were created using SigmaPlot 12.0 (Systat Software Inc., San Jose, CA, USA). All primers used in the present study are listed in Table S1.

## 3. Results

### 3.1. Characterization of MaERF012

**Figure 2:**
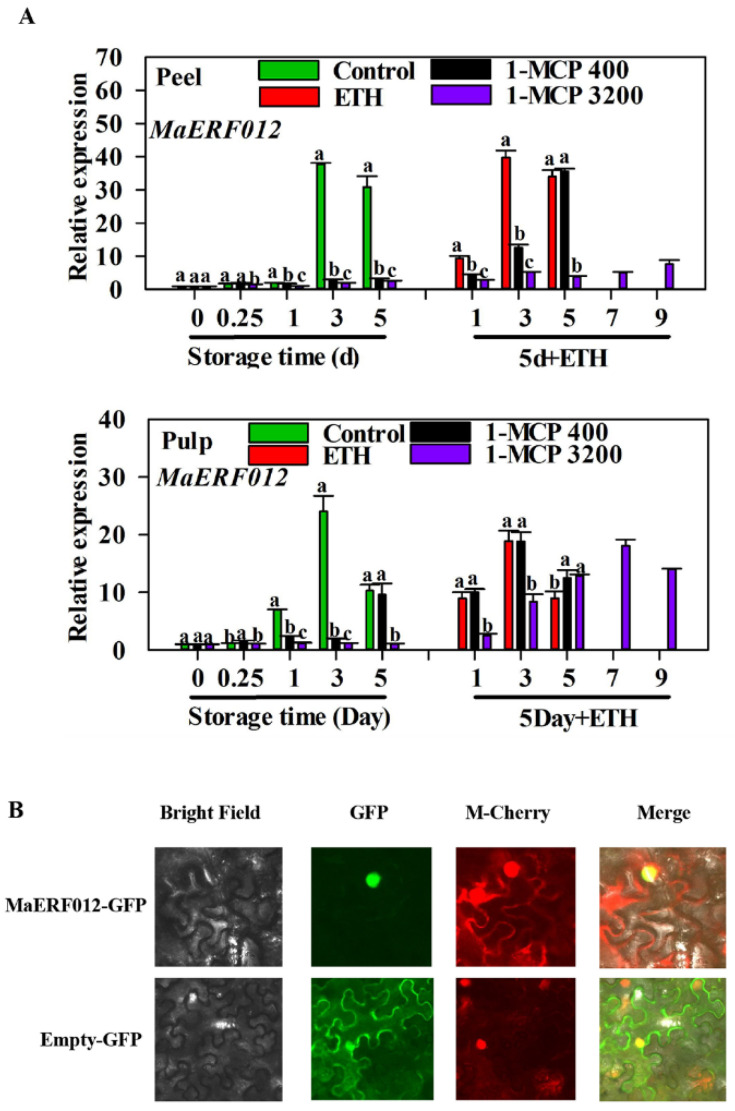
The expression profile and subcellular localization of *MaERF012*. (**A**) The expression of *MaERF012* in fruit peel and pulp under different treatments during storage. Different letters indicate significant difference (*p* < 0.05). (**B**) Subcellular localization of *MaERF012*. MaERF012-GFP and the positive controls pEAQ-GFP and M-Cherry were transiently expressed in tobacco leaves. The images were obtained using a fluorescence microscope with green fluorescence. Bars = 50 μm.

As shown in Figure 1, different 1-MCP treatments were employed in the present study, of which suitable 1-MCP treatment inhibited fruit ripening, while a high concentration of 1-MCP caused a ripening disorder in ‘Fenjiao’ banana fruits, as described in a previous study [3]. The transcript level of *MaERF012* increased with fruit ripening, but 1-MCP treatment significantly repressed its expression in ‘Fenjiao’ banana pulp and peel during storage. In the peel and pulp, the transcription of *MaERF012* in the suitable 1-MCP treatment could recover from ethephon treatment after 5 days of storage. However, in the high-concentration 1-MCP treatment, the expression of *MaERF012* could recover from ethephon treatment in the pulp but not in the peel after 5 days of storage (Figure 2A), which was similar to our previous study [3].

As shown in Figure S1, we found that MaERF012 protein contained 188 amino acids and an AP2/ERF domain. To understand which group MaERF012 belongs to, tomato, papaya, and other banana ERF proteins were used for phylogenetic analysis. As shown in Figure S2, MaERF012 was closely related to SlERF014. Additionally, we found that MaERF012 was located in the cell nucleus (Figure 2B).

### 3.2. MaERF012 Activates the Transcription Activity of Chl, Starch, and Cell Wall Degradation Genes

**Figure 3:**
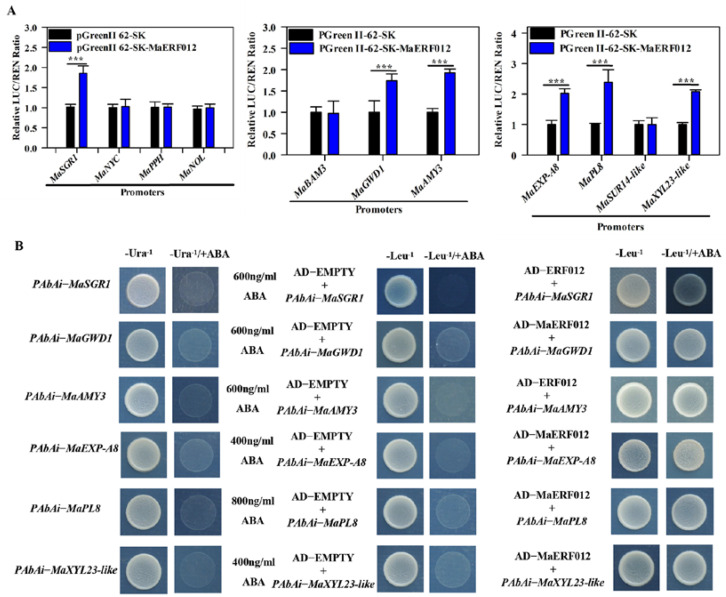
MaERF012 activates the promoter activity of Chl, starch, and cell wall degradation-related genes. (**A**) DLR was used to assay the LUC activity of four Chl degradation, three starch degradation, and four cell wall degradation genes regulated by MaERF012. An empty vector was used as a reference control (set as 1). *** indicates significantly different at *p* < 0.001 level. (**B**) Y1H was used to assay the interaction between MaERF012 and the promoter of *MaSGR1*, *MaGWD1*, *MaAMY3*, *MaEXP-A8*, *MaPL8*, and *MaXYL23-like*.

**Figure 4:**
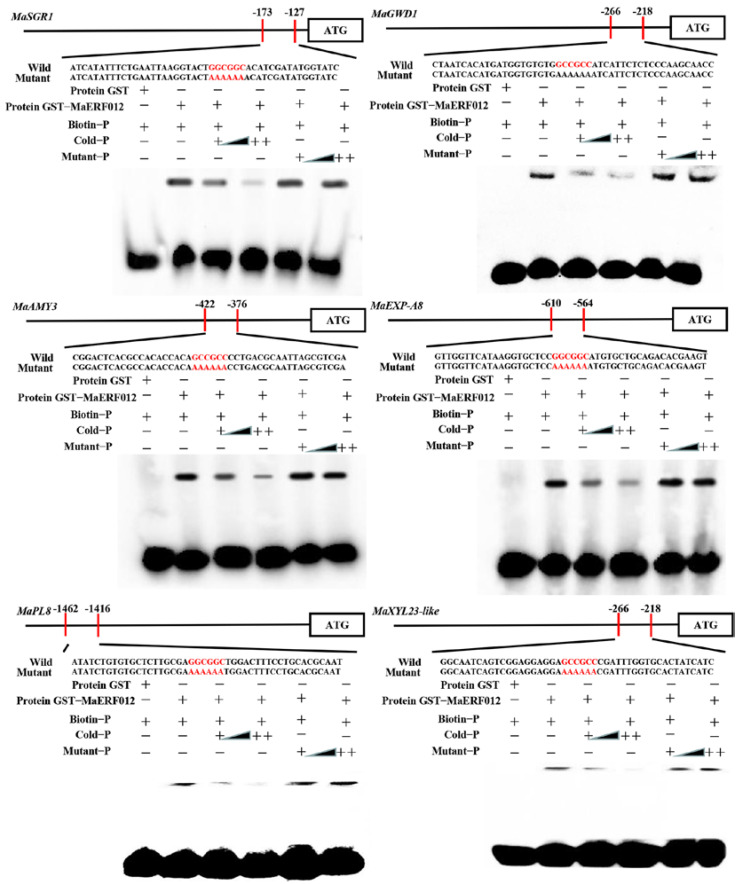
MaERF012 binds to the promoters of Chl, starch, and cell wall degradation genes in vitro. The interaction of MaERF012 and the promoter of *MaSGR1*, *MaGWD1*, *MaAMY3*, *MaEXP-A8*, *MaPL8,* and *MaXYL23-like* was verified using an EMSA. GST-MaERF012 was mixed with the probes for *MaSGR1*, *MaGWD1*, *MaAMY3*, *MaEXP-A8*, *MaPL8,* and *MaXYL23-like* promoters including the GCC-box motifs and the mutant probe AAAAAA, which is shown with red letters. ‘−’ represents absence; ‘+’ represents presence; ‘++’ indicates increasing amounts of unlabeled or mutated probes for competition.

The promoter fragments of four Chl degradation-related genes (*MaSGR1*, *MaNYC*, *MaPPH*, and *MaNOL*), three starch degradation-related genes (*MaBAM3*, *MaGWD1*, and *MaAMY3*) and four cell wall degradation-related genes (*MaEXP-A8*, *MaPL8*, *MaSUR14-like*, and *MaXYL23-like*) containing the GCC-box motif were cloned to the reporter vector, and the CDS of *MaERF012* was inserted into the MaERF012-SK effector vector (Figures S3 and S4). As shown in Figure 3A, the LUC activity of *MaSGR1*, *MaGWD1*, *MaAMY3*, *MaEXP-A8*, *MaPL8*, and *MaXYL23-like* was significantly higher (2–3 fold) in the presence of MaERF012 compared to the control group; however, the LUC activity of *MaNYC*, *MaPPH*, *MaNOL*, *MaBMY3,* and *MaSUR14-like* in the presence of MaERF012 was not significantly different compared to the control. These data indicate that MaERF012 enhanced the promoter activity of *MaSGR1*, *MaGWD1*, *MaAMY3*, *MaEXP-A8*, *MaPL8*, and *MaXYL23-like*, while MaERF012 had no effect on the promoter activities of *MaNYC*, *MaPPH*, *MaNOL*, *MaBMY3,* and *MaSUR14-like* [3]. The promoter fragment of *MaSGR1*, *MaGWD1*, *MaAMY3*, *MaEXP-A8*, *MaPL8*, and *MaXYL23-like* was then inserted into pAbAi and introduced into the Y1H Gold strain to detect the basal activity of *MaSGR1*, *MaGWD1*, *MaAMY3*, *MaEXP-A8*, *MaPL8*, and *MaXYL23-like*. No basal activity was found for the *MaSGR1*, *MaGWD1*, *MaAMY3*, *MaEXP-A8*, *MaPL8*, and *MaXYL23-like* promoters in yeast (Figure 3B). AD-MaERF012 was transformed into the Y1H reporter strain, and the *MaSGR1*, *MaGWD1*, *MaAMY3*, *MaEXP-A8*, *MaPL8*, and *MaXYL23-like* promoter grew well in the presence of AbA, suggesting that MaERF012 could bind to the promoter of *MaSGR1*, *MaGWD1*, *MaAMY3*, *MaEXP-A8*, *MaPL8*, and *MaXYL23-like* (Figure 3B). Moreover, an EMSA was performed to verify the interaction. The GST-MaERF012 protein was obtained as shown in Figure S5, which was used for the EMSA assay. The findings indicate that MaERF012 interacts with the promoter fragment of *MaSGR1*, *MaGWD1*, *MaAMY3*, *MaEXP-A8*, *MaPL8*, and *MaXYL23-like*. In addition, MaERF012 cannot bind to the promoter fragment when GGCGGC mutates to AAAAAA (Figure 4). 

### 3.3. Overexpression of MaERF012 Promotes ‘Fenjiao’ Banana Ripening 

**Figure 5:**
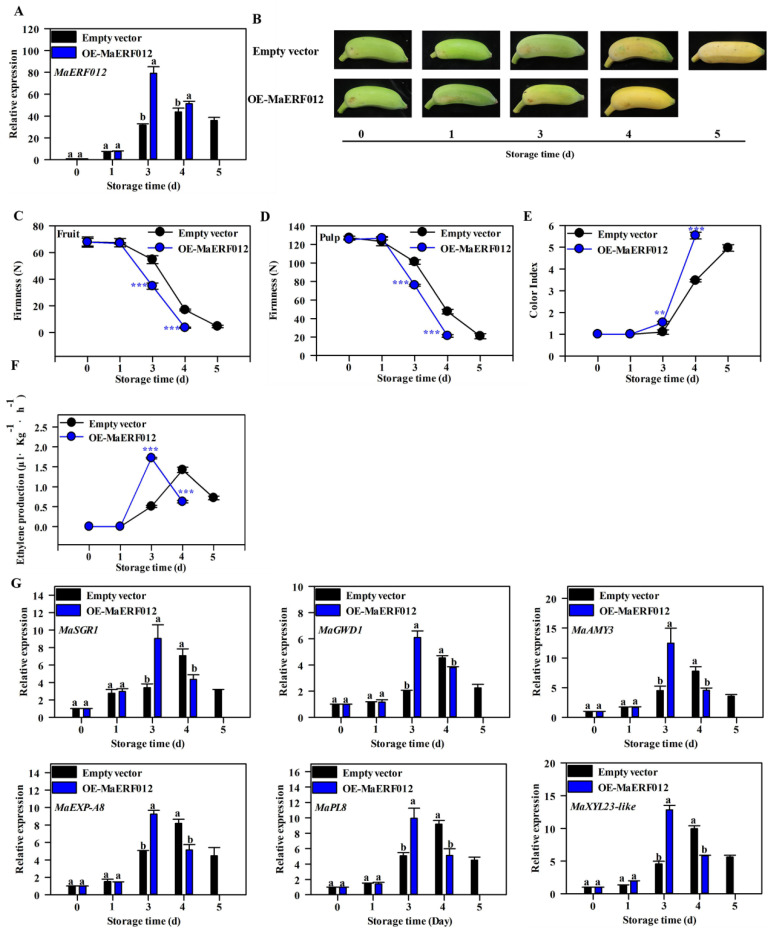
*MaERF012* overexpression promotes fruits ripening in ‘Fenjiao’ banana. (**A**) In ‘Fenjiao’ banana, the expression of *MaERF012* for *MaERF012*-overexpressing and empty vector lines was measured by RT-qPCR. The expression data for the empty vector was set as 1. Different letters indicate significant difference (*p* < 0.05). (**B**) ‘Fenjiao’ banana ripening process of *MaERF012*-overexpressing and empty vector lines. (**C**–**F**) Changes in fruit (**C**) and pulp (**D**) firmness, color index (**E**), and ethylene production (**F**) during fruit ripening. Asterisks (** and ***) indicate significant differences at a level of *p* < 0.01 and *p* < 0.001, respectively. (**G**) *MaERF012* overexpression activates the expression of one Chl degradation, two starch degradation, and three cell wall degradation genes. Different letters indicate significant difference (*p* < 0.05).

To further verify whether MaERF012 mediates fruit ripening by modulating Chl, cell wall, and starch degradation, transient overexpression of *MaERF012* was performed in ‘Fenjiao’ banana. As shown in Figure 5A, the overexpression of *MaERF012* significantly increased the transcript level of *MaERF012*, which suggests that an *MaERF012*-overexpressing (OE) line was successfully created for ‘Fenjiao’ banana. The overexpression of *MaERF012* markedly promotes fruit peel coloring from green to yellow and accelerates fruit softening, ethylene production, and color change compared to the empty vector control (Figure 5B–F). The expression of *MaSGR1* in ‘Fenjiao’ banana peel and the transcription of *MaGWD1*, *MaAMY3*, *MaEXP-A8*, *MaPL8*, and *MaXYL23-like* in ‘Fenjiao’ banana pulp was significantly induced in *MaERF012*-overexpressing lines compared to the control (Figure 5G).

### 3.4. Transcriptomic Analysis of MaERF012 Overexpression in ‘Fenjiao’ Banana

**Figure 6:**
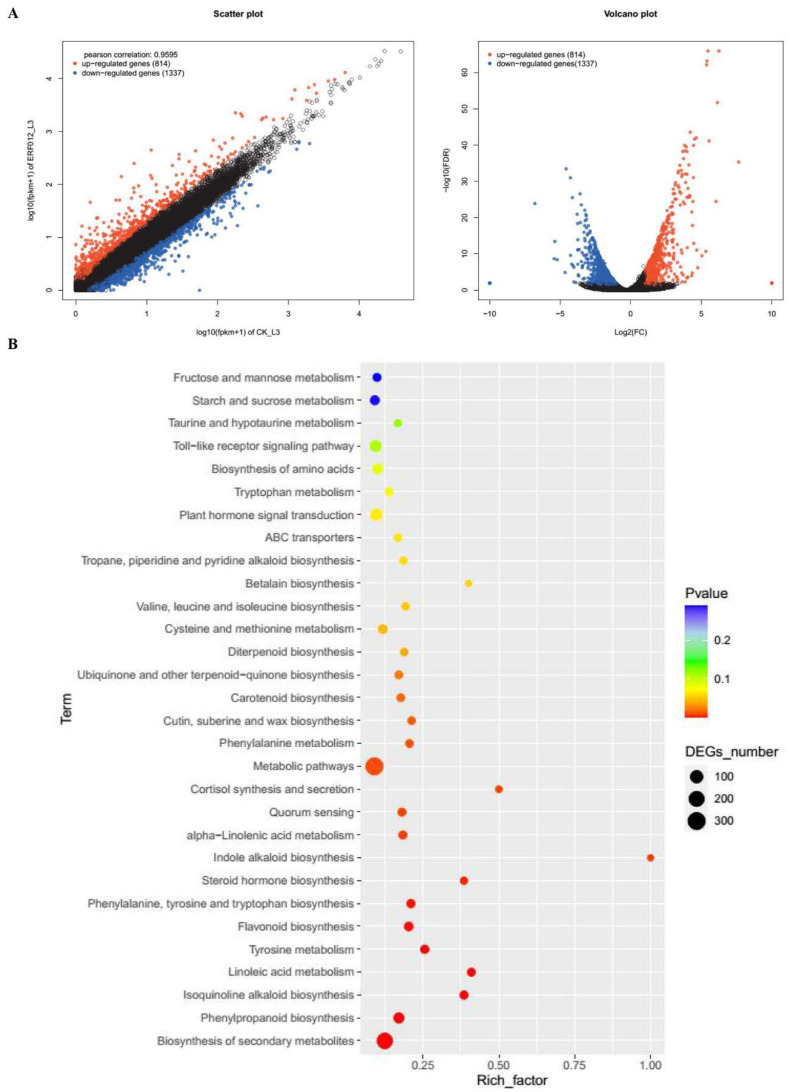
Comparison of DEGs for the *MaERF012*-overexpression and WT lines in fruit peel. (**A**,**B**) volcano plot (**A**) and KEGG enrichment (**B**) of the DEGs identified across WT and *MaERF012*-overexpression lines.

**Figure 7:**
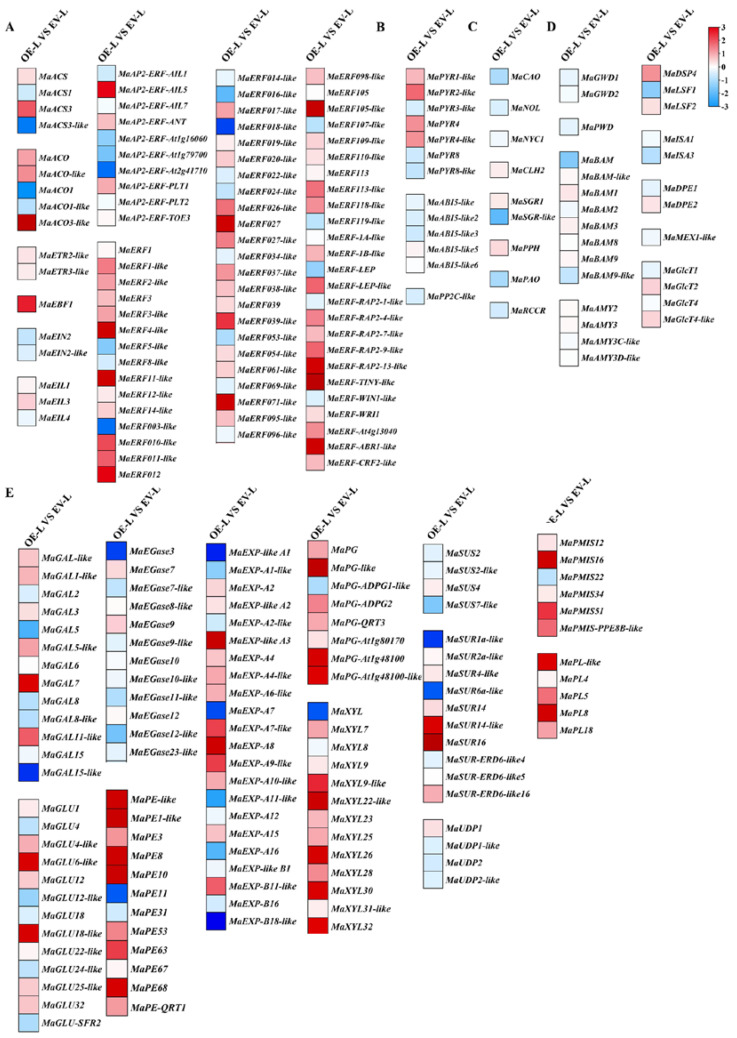
The expression profiles of the key DEGs for different pathways involved in fruit ripening in WT and *MaERF012*-overexpression lines in peel using a heatmap. (**A**–**E**) DEGs in ethylene synthesis and the signal pathway (**A**), the ABA signal pathway (**B**), Chl degradation-related genes (**C**), starch degradation-related genes (**D**), and cell wall degradation-related genes (**E**). Transcription data were taken from RNA-seq and the heatmap was drawn using TBTools. The red and blue color indicated the up- or down-regulation, respectively.

As shown in Figure 5, the overexpression of *MaERF012* significantly promotes fruit ripening. To obtain a global overview of the expression changes in *MaERF012*-overexpressing fruits, we analyzed the DEGs across *MaERF012*-overexpressing and control lines with RNA-Seq. Figure 6A shows that 2151 DEGs were identified in *MaERF012*-overexpressing and control lines in ‘Fenjiao’ banana peel, including 814 up-regulated genes and 1337 down-regulated genes. Among the top 30 enriched metabolic pathways, the DEGs were most enriched in metabolic pathways, biosynthesis of secondary metabolites, phenylpropanoid biosynthesis, flavonoid biosynthesis, starch and sucrose metabolism, and plant hormone signal transduction (Figure 6B). We further analyzed the expression profiles and signaling pathway of ethylene synthesis, the ABA signaling pathway, and Chl, cell wall, and starch degradation-related genes. As shown in Figure 7, the overexpression of *MaERF012* induced the transcription of most genes associated with ethylene synthesis and its signaling pathway, the ABA signaling pathway, and Chl, cell wall, and starch degradation in ‘Fenjiao’ banana peel. Specifically, *MaACS*, *MaACO*, *MaEBF1*, and *MaERF4-like* had increased transcription in ethylene synthesis and the signaling pathway. *MaPYL4-like* and *MaABI5-like* showed increased transcription in the ABA signaling pathway. *MaSGR1* and *MaPPH* showed increased transcription in Chl degradation. *MaBAM3* and *MaAMY3* had increased transcription in the starch signaling pathway, and *MaEXP-A8*, *MaPL8*, and *MaXYL23* had increased transcription in cell wall degradation. 

**Figure 8:**
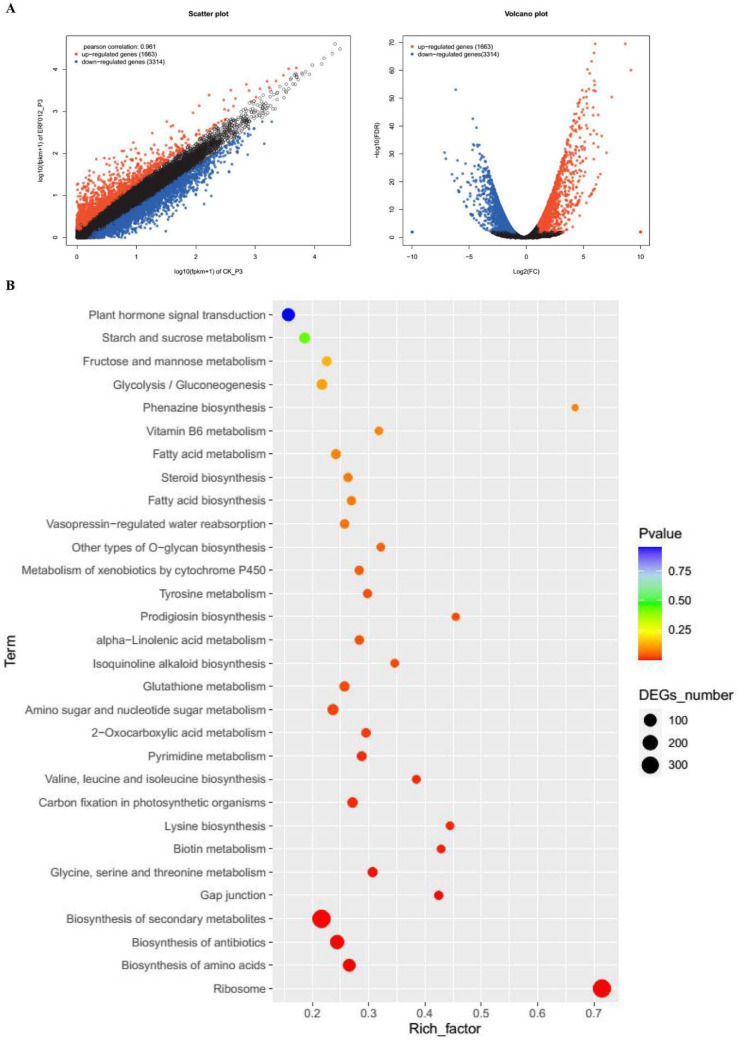
Comparison of the DEGs identified in *MaERF012*-overexpression and WT lines in fruit pulp. (**A**,**B**) volcano plot (**A**) and KEGG enrichment (**B**) of the DEGs identified across WT and *MaERF012*-overexpression lines.

**Figure 9:**
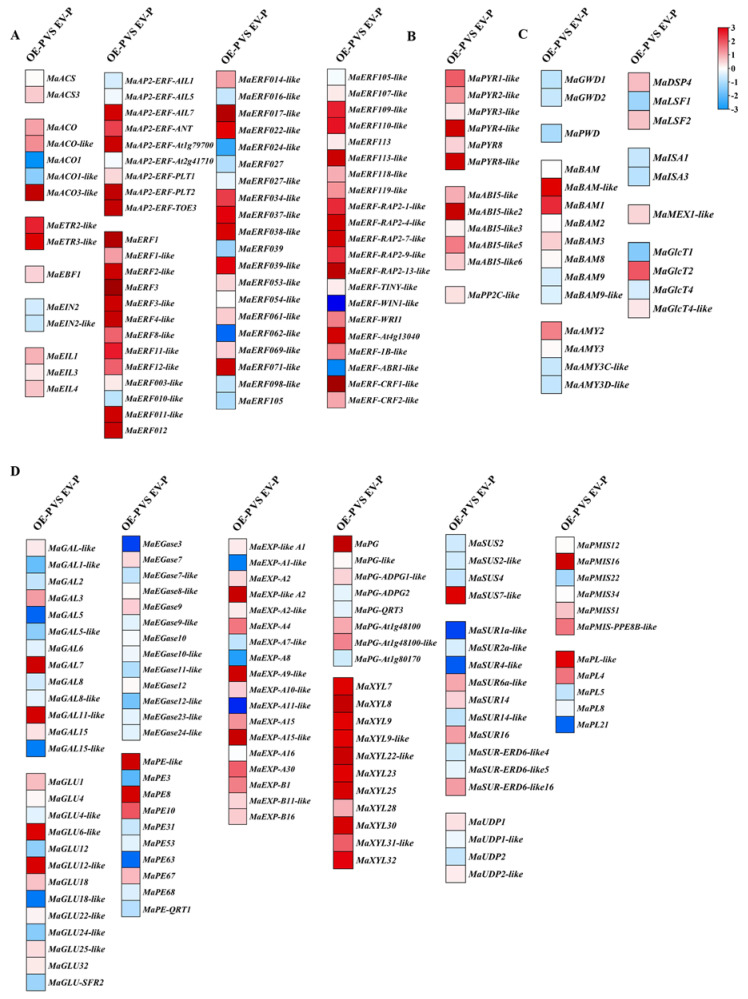
The expression profiles of the key DEGs in *MaERF012*-overexpression and WT lines in fruit pulp. (**A**–**D**) DEGs in ethylene synthesis and its signal pathway (**A**), the ABA signal pathway (**B**), starch degradation-related genes (**C**), and cell wall degradation-related genes (**D**). Transcription data were taken from RNA-seq and the heatmap was drawn using TBTools. The red and blue color indicated the up- or down-regulation, respectively.

Moreover, as shown in Figure 8A, 4977 DEGs were identified across *MaERF012*-overexpressing and control lines in ‘Fenjiao’ banana pulp, including 1663 up-regulated genes and 3314 down-regulated genes. Among the top 30 metabolic pathways, the DEGs were most enriched in the biosynthesis of secondary metabolites, the biosynthesis of antibiotics, the biosynthesis of amino acids, starch and sucrose metabolism, plant hormone signal transduction, and fructose and mannose metabolism pathways (Figure 8B). The expression profiles of ethylene synthesis and the signaling pathway, the ABA signaling pathway, and cell wall and starch degradation-related genes were also analyzed in ‘Fenjiao’ banana pulp. As shown in Figure 9, the overexpression of *MaERF012* induced the transcription of most genes associated with ethylene synthesis and signaling, ABA signaling, and starch and cell wall degradation. Specifically, *MaACS3*, *MaACO*, and *MaEBF1* in ethylene synthesis and its signaling pathway showed up-regulation. *MaPYL8-like* and *MaABI5-like* were up-regulated in the ABA signaling pathway. *MaBAM1* and *MaAMY2* showed increased expression in the starch signaling pathway. *MaEXP-A8*, *MaPL8*, and *MaXYL23* had increased expression in cell wall degradation. However, the expression of *MaGWD1* was repressed in RNA-Seq data. 

## 4. Discussion

Yellowing and softening are two vital indicators of banana fruit ripening, which are co-determined due to Chl, cell wall, and starch degradation [6,9]. ‘Fenjiao’ banana fruit accumulates an abundance of starch in the cell wall to maintain banana firmness, and the fruit was harvested at an 85–90% maturity stage in terms of green peel [9,27]. During fruit ripening, yellowing correlates with Chl degradation [5], and softening is closely associated with starch and cell wall degradation [9]. ‘Fenjiao’ bananas are a climacteric fruit, ripening quickly due to peak ethylene production after harvesting [12]. Treatments involving 1-MCP have been widely used in flowers, vegetables, and fruits to prolong shelf-life [33,34]. Our previous study found that the use of a low-concentration 1-MCP treatment effectively prolongs storage time for fruit, allowing fruit to ripen normally after ethephon treatment. However, the use of a high concentration of 1-MCP caused a ripening disorder (fruit pulp can soften but the peel does not turn yellow), even after treatment with ethephon [3].

The ERF TF family, a downstream component of the ethylene signaling pathway, has been reported to modulate plant growth, stress response, and fruit development and ripening [17,35]. However, the mechanisms by which most ERFs modulate climacteric fruit ripening are still unknown. Herein, we demonstrated that the expression of *MaERF012* was induced with fruit ripening and inhibited by 1-MCP treatment (suitable and unsuitable treatment). However, the expression of *MaERF012* could recover under suitable 1-MCP treatment followed by ethephon treatment in both the pulp and peel. Under the high-concentration 1-MCP treatment, the expression of *MaERF012* in fruit peel did not recover following ethephon treatment (Figure 2). The uneven expression of *MaERF012* in fruit peel and pulp may be due to a fruit ripening disorder partially induced by high-concentration 1-MCP treatment. The results indicate that MaERF012 may modulate a fruit ripening disorder caused by the high-concentration 1-MCP treatment, which is consistent with our previous work [3].

Several ERFs have been shown to be involved in Chl degradation. PyERF3 interacts with PybHLH3 and PyMYB114 proteins to form protein complexes that mediate red coloration in pear fruit [36]. In citrus, CitERF13 and CitERF6 modulate Chl degradation in fruit by directly activating the transcription of *CitPPH* [37,38]. In lemon, ClERF114 may regulate fruit Chl degradation by modulating *ClNCED5*, *ClPPH*, and *ClCLH1* [39]. In tomato, SlERF16 promotes fruit degreening by activating the transcription of *SlPPH* genes [38]. The reduced expression of *SlERF.F12* represses tomato fruit ripening and color changes [40]. Four Chl degradation genes (*MaPPH1*, *MaNCY1*, *MaNOL*, and *MaSGR1*) were previously identified and found to be closely associated with a fruit ripening disorder caused by high-concentration 1-MCP treatment [3]. Herein, we found that the promoters of *MaPPH1*, *MaNCY1*, *MaNOL*, and *MaSGR1* contained a GCC-box, which may be modulated by ERFs. In addition, MaERF012 can directly interact with the *MaSGR1* promoter by binding the GCC-box motif. Additionally, the promoter activity of *MaSGR1* was induced by MaERF012 (Figure 3 and Figure 4). The high-concentration 1-MCP treatment severely inhibited the transcription of *MaERF012*, and the transcription of *MaERF012* could not recover after ethephon treatment in the peel, which may be the main cause of abnormal yellowing in ‘Fenjiao’ bananas.

Several studies have previously indicated that ERF TFs also regulate fruit softening by modulating starch and cell wall degradation. In kiwifruit, AdERF9 represses the transcription level of the cell wall degradation gene *AdXET5* during fruit ripening [41]. PpeERF2 inhibited cell wall degradation by inhibiting the transcriptional activation activity of *PpePG1* in peach [26]. In papaya, CpERF9 modulates cell wall degradation by inhibiting the promoter activity of *CpPG5* and *CpPME1/2* [20]. In tomato, the overexpression of *SlERF.B3* represses the onset of ripening, improves ethylene production, and promotes fruit softening [17,18]; *SlERF.F12* represses the expression of *SlPG2a* and *SlPL* by directly interacting with their promoter sequence in banana [39]. Additionally MaERF9 modulates fruit softening by regulating eight cell wall degradation-related genes (*MaPL2*, *MaPME3*, *MaPG1*, *MaEXP1/2/3/5*, and *MaXET7*) [42]. Our previous work identified three starch and four cell wall degradation genes [3] that contain a GCC-box motif in their promoter sequence. The present study found that MaERF012 can directly bind to the GCC-box motif in the promoters of *MaGWD1*, *MaAMY3*, *MaEXP-A8*, *MaPL8*, and *MaXYL23-like*. The promoter activity of these genes was induced by MaERF012 (Figure 3 and Figure 4). Although the high-concentration 1-MCP treatment markedly inhibited the transcription of *MaERF012* in pulp, the transcription of *MaERF012* was able to recover after ethephon treatment; thus, the expression level of genes associated with starch and cell wall degradation were able to recover to a normal level, allowing the pulp of ‘Fenjiao’ banana to normally soften. These results support that MaERF012 is involved in modulating the fruit softening process.

Additionally, the transient overexpression of *MaERF012* promotes fruit ripening, including fruit degreening and softening, by up-regulating the transcription of Chl, starch, and cell wall degradation genes (Figure 5, Figure 7 and Figure 9). However, the expression of *MaGWD1* was repressed in RNA-Seq and induced in RT-qPCR. The difference between RNA-Seq and RT-qPCR may have been caused by a miscalculation regarding FPKM in RNA-Seq, likely caused by the presence of rRNA or other species contamination in the sample. Moreover, some genes were only expressed in pulp, not in peel, including *MaERF62-like*, *MaCRF1-like*, *MaEGase24-like*, *MaEXP-A15-like*, *MaEXP-A30*, *MaEXP-B1,* and *MaPL21*; conversely, some genes were only expressed in peel, not in pulp, including *MaACS1*, *MaACS3-like*, *MaAP2-ERF-At1g16060*, *MaERF5-like*, *MaERF14-like*, *MaERF18-like*, *MaERF19-like*, *MaERF20-like*, *MaERF26-like*, *MaERF95-like*, *MaERF96-like*, *MaERF-1A-like*, *MaERF-LEP*, *MaERF-LEP-like*, *MaPYL4*, *MaDPE1*, *MaDPE2*, *MaPE1-like*, *MaPE11*, *MaEXP-like A3*, *MaEXP-A4-like*, *MaEXP-A6-like*, *MaEXP-A7*, *MaEXP-A12*, *MaEXP-like B1*, *MaEXP-B18-like*, *MaXYL*, *MaXYL26,* and *MaPL18*. In addition, the expression profiles of some genes in *MaERF012*-overexpressing pulp were different to *MaERF012*-overexpressing peel. Compared to the control, many genes were enhanced in *MaERF012*-overexpressing pulp but inhibited in *MaERF012*-overexpressing peel, including *MaEIL4*, *MaAP2-ERF-At1g79700*, *MaERF8-like*, *MaERF003-like*, *MaERF014-like*, *MaERF052*, *MaERF107-like*, *MaERF119-like*, *MaPYR3-like*, *MaPYR8*, *MaPYR8-like*, *MaABI5-like*, *MaABI5-like2*, *MaABI5-like3*, *MaPP2C-like*, *MaMEX1-like*, *MaGLU18*, *MaEXP-like A1*, *MaEXP-A2-like*, *MaEXP-B16*, *MaPG-ADPG1-like*, *MaXYL8*, *MaSUS7-like, MaSUR6a-like,* and *MaUDP2-like*. Meanwhile, compared to control, many genes were repressed in *MaERF012*-overexpressing pulp but enhanced in *MaERF012*-overexpressing peel, including *MaAP2-ERF-AIL5*, *MaERF10-like*, *MaERF027*, *MaERF027-like*, *MaERF039*, *MaERF098-like*, *MaERF-ABR1-like*, *MaBAM9*, *MaGAL1-like*, *MaGAL5-like*, MaGLU4-like, *MaGLU12*, *MaGLU18-like*, *MaPE3*, *MaPE53*, *MaPE63*, *MaPE68*, *MaPE-QRT1*, *MaEXP-A7-like*, *MaEXP-A8*, *MaPG-like*, *MaPG-ADPG2*, *MaPG-QRT3*, *MaPG-At1g80170*, *MaSUS4*, *MaSUR2a-like*, *MaSUR14-like*, *MaPL5,* and *MaPL8*. These data indicate that *MaERF012* overexpression differentially affects the expression of some genes in pulp and peel.

The overexpression of MaERF012 alters the transcriptome profiles of the fruit peel and pulp, and the DEGs were mainly enriched in the biosynthesis of secondary metabolism, fructose and mannose metabolism, starch and sucrose metabolism, and plant hormone signal transduction. Taken together, our data showed that MaERF012 acts as a transcriptional activator and regulates fruit ripening by activating the transcription of Chl, starch, and cell wall degradation genes. These data further indicated that MaERF012 modulates fruit degreening and softening, hence playing a role in the ripening of ‘Fenjiao’ banana.

## 5. Conclusions

**Figure 10:**
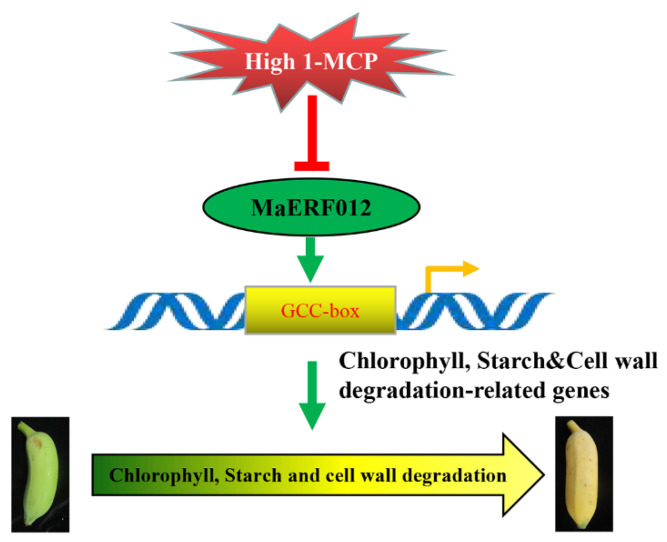
The proposed working model of MaERF012 regulating chlorophyll, starch, and cell wall degradation.

Overall, this study identified MaERF012 as a new transcriptional activator with the role of activating the transcriptional activity of genes associated with Chl, starch, and cell wall degradation by directly interacting with their promoters. Moreover, transient overexpression of *MaERF012* promotes the ripening of fruit by promoting Chl, starch, and cell wall degradation in fruit. However, under the high-concentration 1-MCP treatment, the expression of *MaERF012* was significantly repressed, which then markedly repressed the expression of genes related to Chl, starch, and cell wall degradation that are regulated by MaERF012. The high-concentration 1-MCP treatment caused an uneven expression of MaERF012 in fruit peel and pulp after ethephon treatment, which significantly inhibited the transcription of *MaERF012* in the peel and pulp; the transcription of *MaERF012* was able to recover after ethephon treatment in the pulp but not in the peel (Figure 10). This uneven expression of MaERF012 affected the degreening and fruit softening process and caused a ripening disorder in ‘Fenjiao’ banana. Together, our results revealed that the transcriptional regulatory network of MaERF012 is involved in Chl, starch, and cell wall degradation during fruit ripening in ‘Fenjiao’ banana.

## Data Availability

The date are available from the corresponding author.

## Supplementary Materials

The following supporting information can be downloaded at: https://www.mdpi.com/article/10.3390/foods11233882/s1, Figure S1. Multiple alignment of MaERF012 (XM_009415777.2) protein with other ERF proteins, Figure S2. Phylogenetic analysis of MaERF012 and other ERF proteins, Figure S3. Picture of the effector and reporter vectors, Figure S4. The location of GCC-box motifs in the promoters of three Chl degradation, three starch, and four cell wall degradation genes, Figure S5. The recombinant proteins of GST-MaERF012 were tested by gel-stained SDS-PAGE, Table S1. Primers used in the present study.
