# Supplementary material for: Ethylene Response Factor MaERF012 Modulates Fruit Ripening by Regulating Chlorophyll Degradation and Softening in Banana
Source: Foods. 2022 Dec 1;11(23):3882. doi: 10.3390/foods11233882 (PMC9738063; doi:10.3390/foods11233882)
Supplement: Supplementary file 1 [file foods-11-03882-s001.zip › foods-2042579-supplementary.pdf]

Supplementary Materials

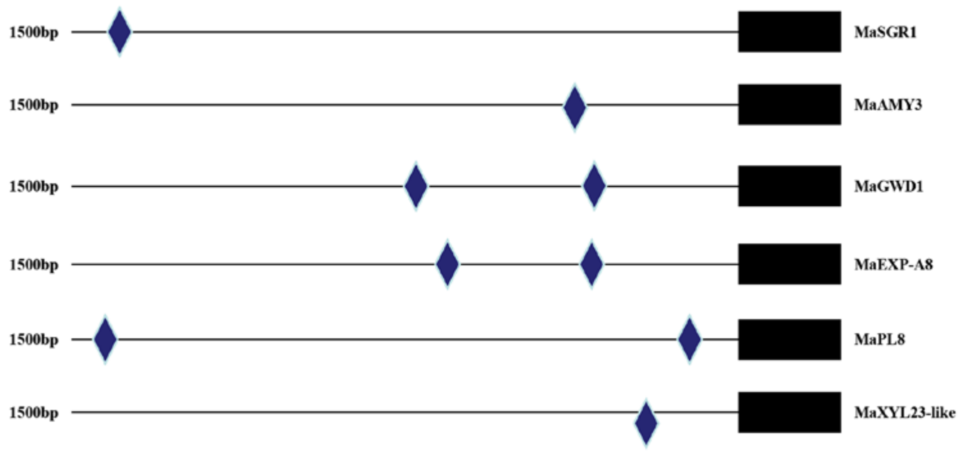

Figure S1. Multiple alignment of MaERF012 (XM_009415777.2) protein with other ERF proteins. Other plants ERF proteins including banana MaERF003 (XP_009417483.2) and MaERF113 (XP_018683838.1), tomato SlERF003 (XP_004242199.1), SlERF014 (XP_010312777.1), SlERF017 (XP_004251702.1) and SlERF021 (XP_004250714.1), papaya CpERF003 (XP_021906523.1), CpERF113 (XP_021900277.1), CpERF020 (XP_021903568.1), CpERF023 (XP_021893066.1), CpERF024 (XP_021908529.1), CpERF036 (XP_021891938.1) and CpERF039 (XP_021893663.1). The multiple alignments were made using DNAMAN.

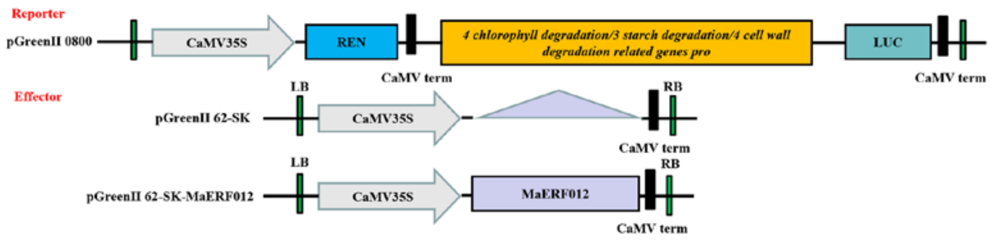

Figure S2. Phylogenetic analysis of MaERF012 and other ERF proteins. The phylogenetic tree was performed by MEGA 5.0 with a default parameters, minimum evolution test and bootstrap test of phylogeny. The numbers indicate the bootstrap values.

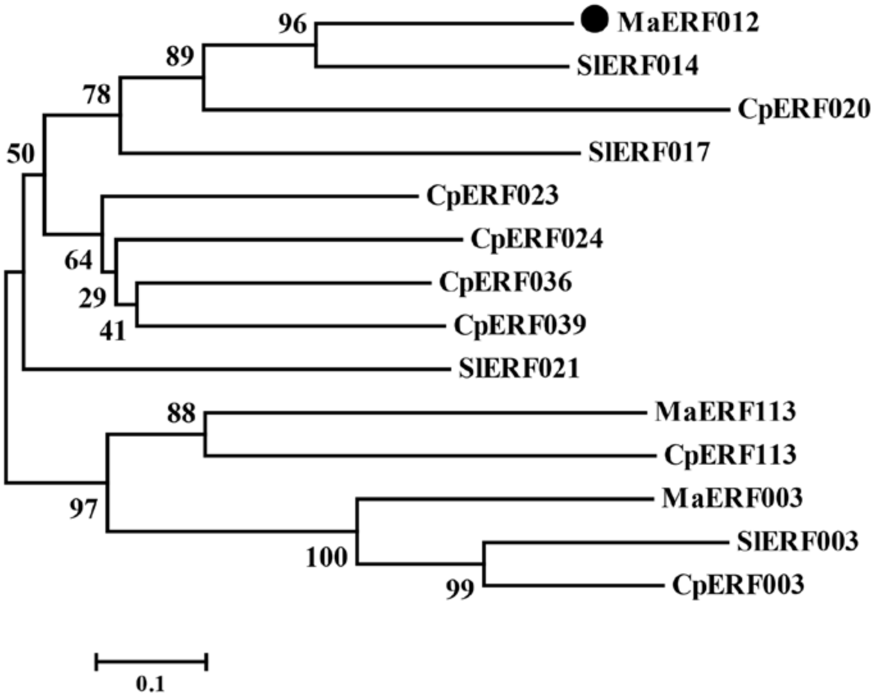

Figure S3. Picture of the effector and reporter vectors.

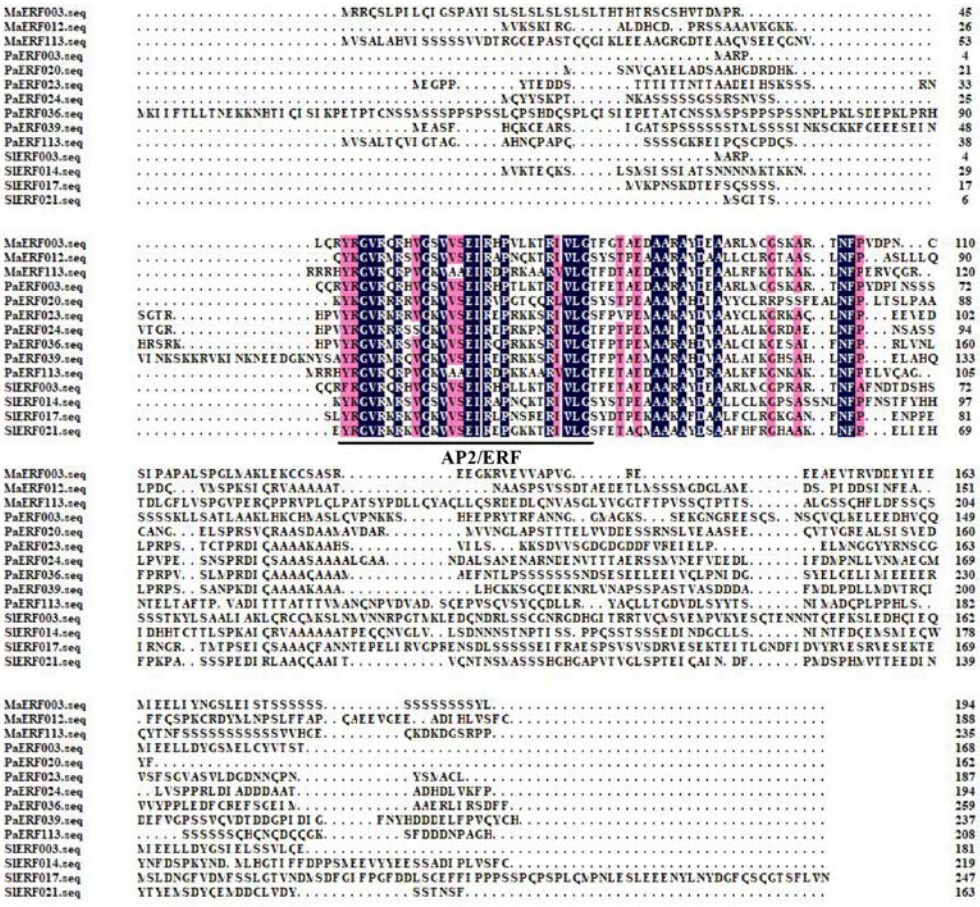

Figure S4. The location of GCC box motifs in the promoters of 3 Chl degradation, 3 starch and 4 cell wall degradation genes.

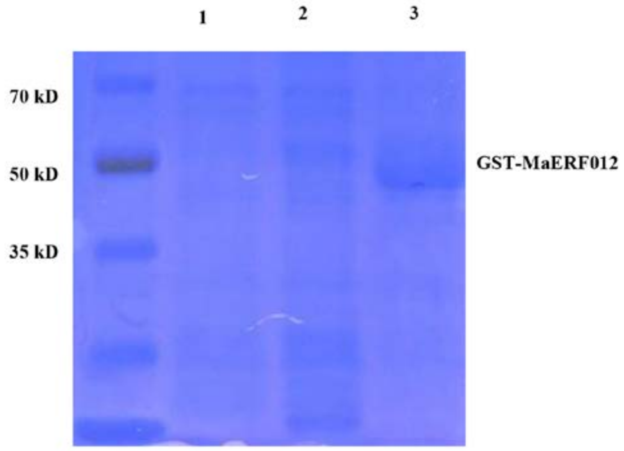

Figure S5. The recombinant proteins of GST-MaERF012 was tested by SDS-PAGE gel stained. In lane 1-3: non-induced protein, before purification of the induced protein, after purification of the induced protein.

Table S1. Primers used in the present study.

Experiment	Gene	Forward primer (5′-3′)
RT-qPCR	*MaERF012-F*	AGACCTTCTTCCAGTCACCCA
	MaERF012-R	GAGCAATCTACTTGACTTCCACC
	MaSGR1-F	GGAACAAGGTCCAAGGAAAGA
	MaSGR1-R	GGAGTTCATCGCAGAAGATGTA
	MaGWD1-F	GTGTAAAGGGAGAGGAGGAAATAC
	MaGWD1-R	CTCGCACAGAGACATGAGATAG
	MaAMY3-F	AGGAACAGGCTCTGGGTATG
	MaAMY3-R	AGACTCAGTGGGTGGTGGTA
	MaPL8-F	AGGTTCTCACAGGTTGTTGT
	MaPL8-R	TCTCTTCCAAGGAAGCATCG
	MaEXP-A8-F	GCTCTGGGAGATGATTGGATT
	MaEXP-A8-R	CGTCGGTGTACAAGTTCTGATA
	MaXYL23-like-F	GTCACCGCCTACTACCTATCT
	MaXYL23-like-R	TTGGTGTGCAGAGTGTAAGG
Subcellular location	ERF012-like-GFP-F	ATCAAGCTTGGATCCATGGTGAAGAGCAAGATCAG
	ERF012-like-GFP-R	GCTCACCATGTCGACGCAGAAGCTCCACAG
Transient expression assay	MaERF012-like-sk-F	CGCTCTAGAACTAGTGGATCCATGGTGAAGAGCAAGATCAG
	MaERF012-like-sk-R	GATAAGCTTGATATCGAATTCTCAGCAGAAGCTCCACAG
	MaNOL-0800-F	CTATAGGGCGAATTGGGTACCATCTATTATTACACATGC
	MaNOL-0800-R	TGTTTTTGGCGTCTTCCATGGGACTAGAATAAGCTTATAT
	MaNYC1-0800-F	CTATAGGGCGAATTGGGTACCACCGTGAACAATTTTGGATGC
	MaNYC1-0800-R	TGTTTTTGGCGTCTTCCATGGGATGCTTGAAATCTGCGCATGT
	MaSGR1-0800-F	CTATAGGGCGAATTGGGTACCACTTCTCTTAGCATGAGC
	MaSGR1-0800-R	TGTTTTTGGCGTCTTCCATGGGTACTTAAACTTCCGAGGGAT

	MaPPH-0800-F:	CTATAGGGCGAATTGGGTACCTGTGAAGGTGCCCTACTG
	MaPPH-0800-R:	TGTTTTTGGCGTCTTCCATGGCATGATCGTGTCGCCCTT
	MaBAM3-0800-F	CTATAGGGCGAATTGGGTACCATTGCCTCCAGTACCTAACATTCAG
	MaBAM3-0800-R	TGTTTTTGGCGTCTTCCATGGCATACTGTCGGAGGCCGGCGA
	MaGWD1-0800-F	CTATAGGGCGAATTGGGTACCAATGACCCCATTAGATCGGATC
	MaGWD1-0800-R	TGTTTTTGGCGTCTTCCATGGACTTTCGTTGGAGGGACATCC
	MaAMY3-0800-F	CTATAGGGCGAATTGGGTACCTCCTTCTGCGTTAGCCGTTG
	MaAMY3-0800-R	TGTTTTTGGCGTCTTCCATGGCGGCGAACGGTGGGGGAGAATC
	MaPL8-0800-F	CTATAGGGCGAATTGGGTACCTCGTATCACCATTCGCACCTG
	MaPL8-0800-R	TGTTTTTGGCGTCTTCCATGGGATGGAGAATGCAGTTGCAAGG
	MaEXP-A8-0800-F	CTATAGGGCGAATTGGGTACCTAGGACCACTTTCATGCCTACAG
	MaEXP-A8-0800-R	TGTTTTTGGCGTCTTCCATGGGCTGTGAATATTAATGCATACCATC
	MaSUR14-like-0800-F	CTATAGGGCGAATTGGGTACCTTAATGGGCCATTAAACCTTGACAG
	MaSUR14-like-0800-R	TGTTTTTGGCGTCTTCCATGGCTCTTTCTTCCTCCTCTTTCAAG
	MaXYL23-like-0800-F	CTATAGGGCGAATTGGGTACCTCTGGTGTCGGCAAACTCACC
	MaXYL23-like-0800-R	TGTTTTTGGCGTCTTCCATGGAGGAGAGGAGATGGCAGAAGC
Y1H	MAERF012-LIKE-AD-F	ATGGCCATGGAGGCCAGTGAATTCATGGTGAAGAGCAAGATCAG
	MAERF012-LIKE-AD-R	TGCAGCTCGAGCTCGATGGATCCCTCAGCAGAAGCTCCACAG
	MASGR1-PABAI-F	TTGAATTCGAGCTCGGTACCTACTGGCGGCACATACTGGCGGCACATACTGGCGGCACA
	MASGR1-PABAI-R:	TGCCTCGAGGTCGACTGTGCCGCCAGTATGTGCCGCCAGTATGTGCCGCCAGTA
	MaAMY3-PAbAi-F	TTGAATTCGAGCTCGGTACCCACAGCCGCCCCTCACAGCCGCCCCTCACAGCCGCCCCT
	MaAMY3-PAbAi-R	ATGCCTCGAGGTCGACAGGGGCGGCTGTGAGGGGCGGCTGTGAGGGGCGGCTGTG
	MaGWD1-PAbAi-F	TTGAATTCGAGCTCGGTACCTGTGGCCGCCATCTGTGGCCGCCATCTGTGGCCGCCATC
	MaGWD1-PAbAi-R	ATGCCTCGAGGTCGACGATGGCGGCCACAGATGGCGGCCACAGATGGCGGCCACA
	MaEXP-A8-PAbAi-F	TTGAATTCGAGCTCGGTACCTCCGGCGGCATGTTCCGGCGGCATGTTCCGGCGGCATGT
	MaEXP-A8-PAbAi-R	ATGCCTCGAGGTCGACACATGCCGCCGGAACATGCCGCCGGAACATGCCGCCGGA
	MaPL8-PAbAi-F	TTGAATTCGAGCTCGGTACCCGAGGCGGCTGGACGAGGCGGCTGGACGAGGCGGCTGGA
	MaPL8-PAbAi-R	ATGCCTCGAGGTCGACTCCAGCCGCCTCGTCCAGCCGCCTCGTCCAGCCGCCTCG
	MaXYL23-like-PAbAi-F	TTGAATTCGAGCTCGGTACCAGGAGCCGCCCGAAGGAGCCGCCCGAAGGAGCCGCCCGA
	MaXYL23-like-PAbAi-R	ATGCCTCGAGGTCGACTCGGGCGGCTCCTTCGGGCGGCTCCTTCGGGCGGCTCCT
EMSA	MaERF012-like-GST-F	GGTTCCGCGTGGATCCATGGTGAAGAGCAAGATCAG
	MaERF012-like-GST-R	AGTCACGATGCGGCCGCGCAGAAGCTCCACAG
	MaSGR1-probe-F	ATCATATTTCTGAATTAAGGTACTGGCGGCACATCGATATGGTATC
	MaSGR1-probe-R	GATACCATATCGATGTGCCGCCAGTACCTTAATTCAGAAATATGAT
	MaAMY3-probe-F	CGGACTCACGCCACACCACAGCCGCCCCTGACGCAATTAGCGTCGA
	MaAMY3-probe-R	TCGACGCTAATTGCGTCAGGGGCGGCTGTGGTGTGGCGTGAGTCCG
	MaGWD1-probe-F	CTAATCACATGATGGTGTGTGGCCGCCATCATTCTCTCCCAAGCAACC
	MaGWD1-probe-R	GGTTGCTTGGGAGAGAATGATGGCGGCCACACACCATCATGTGATTAG
	MaEXP-A8-probe-F	GTTGGTTCATAAGGTGCTCCGGCGGCATGTGCTGCAGACACGAAGT
	MaEXP-A8- probe -R	ACTTCGTGTCTGCAGCACATGCCGCCGGAGCACCTTATGAACCAAC
	MaPL8-probe-F	ATATCTGTGTGCTCTTGCGAGGCGGCTGGACTTTCCTGCACGCAAT
	MaPL8-probe-R	ATTGCGTGCAGGAAAGTCCAGCCGCCTCGCAAGAGCACACAGATAT
	MaXYL23-like-probe-F	GGCAATCAGTCGGAGGAGGAGCCGCCCGATTTGGTGCACTATCATC
	MaXYL23-like-probe-R	GATGATAGTGCACCAAATCGGGCGGCTCCTCCTCCGACTGATTGCC
Transgenic Fenjiao banana fruit	MaERF012-Pdnor-F	AAAAAGCAGGCTTCATGGTGAAGAGCAAGATCAG
	MaERF012-Pdnor-R	CAAGAAAGCTGGGTCTCAGCAGAAGCTCCACAG
